# Occupational outcomes of people with multiple sclerosis during the COVID-19 pandemic: a systematic review with meta-analysis

**DOI:** 10.3389/fpubh.2023.1217843

**Published:** 2023-11-27

**Authors:** Bruno Kusznir Vitturi, Alborz Rahmani, Alfredo Montecucco, Guglielmo Dini, Paolo Durando

**Affiliations:** ^1^Department of Health Sciences, University of Genoa, Genoa, Italy; ^2^Ospedale Policlinico San Martino di Genova IRCCS, Genoa, Italy

**Keywords:** multiple sclerosis, demyelinating diseases, immunomodulators, occupational health, unemployment, public health, COVID-19, work

## Abstract

**Background:**

People with Multiple Sclerosis (PwMS) are vulnerable to unfavorable occupational outcomes and the COVID-19 pandemic brought major consequences on people’s professional lives. In this view, we decided to investigate the occupational outcomes of PwMS during the COVID-19 pandemic.

**Methods:**

We performed a systematic review with meta-analysis searching key terms in four databases. We initially included any peer-reviewed original article that enrolled adult patients with the diagnosis of MS and assessed any occupational variable during the COVID-19 pandemic. There were no time limits and no language restrictions. The primary outcomes were the prevalence of unemployment, retirement and employment status change among people with MS during the COVID-19 pandemic. Other outcomes included the modality and characteristics of work: type of work, full-time work, part-time work and remote work. We also searched for data from studies that addressed any change in the work status due to the COVID-19 outbreak.

**Results:**

We identified 49 eligible articles comprising a total sample size of 17,364 individuals with MS. The pooled prevalence of unemployment and retirement was 0.47 (95% CI = 0.42–0.53). The pooled prevalence of PwMS who were unemployed or retired was positively associated with the progressive phenotype of the disease (*p* = 0.017) and the use of glatiramer acetate (*p* = 0.004), but negatively associated with hospitalization due to COVID-19 (*p* = 0.008) and the use of immunosuppressants (*p* = 0.032), siponimod (*p* < 0.001), and cladribine (*p* = 0.021). The pooled proportion of PwMS that reported any change of the employment status during the COVID-19 pandemic was 0.43 (95% CI = 0.36–0.50) while the pooled prevalence of PwMS who worked remotely during this period was 0.37 (95% CI = 0.15–0.58). The change in employment status was negatively associated with the duration of MS (*p* = 0.03) but positively associated with the progressive phenotype of the disease (*p* < 0.001).

**Conclusion:**

Our seminal review may serve as an example of how patients with neurological diseases or disabilities in general may have their jobs impacted in a pandemic and foster the context of global socio-economic crisis.

## Introduction

Multiple Sclerosis (MS) is an autoimmune disease that affects the central nervous system causing demyelination and neurodegeneration (1). The global prevalence is 35.9 per 100,000 people and an estimated 2.8 million people are living with MS worldwide. Since 2013, its incidence is known to increase annually worldwide, making it an object of great public health interest (2). It represents the leading cause of non-traumatic disability in young people (3). Currently, immunomodulatory drugs are widely available and are considered the first line of treatment for MS (4). Nonetheless, people with MS (PwMS) are still vulnerable to motor, sensory, cognitive, mental, and visual symptoms that offer noticeable impairment to their quality of life (5).

MS is a disease whose onset coincides with the working age and is therefore one of the most impacting diseases in professional life. The great variety of symptoms makes the management of the worker with MS extremely complex (6). Patients with a minimal degree of disability are not spared from the deleterious influence of the disease in many life domains, including the work sphere (7, 8). Only 1 in 6 PwMS do not report any work problems related to the disease (9). PwMS are known to face numerous barriers at work related to the severity of the disability. Moreover, difficulties inherent to any job position that may be easily endured by workers, in general, can be unsustainable for PwMS and may play a crucial role in the risk of unemployment. Similarly, PwMS usually require several types of reasonable accommodations to promote the maximum integration of the worker into the workplace (10). PwMS have higher rates of unemployment and early retirement (11). Indeed, studies showed that once unemployed, this group of patients is unlikely to return to the workforce (12).

The COVID-19 pandemic challenged the resilience of public health systems. Despite the deaths caused by the SARS-CoV-2 infection, the pandemic undermined the possibility of continuing to provide optimal assistance to patients with chronic diseases, particularly those with neurological diseases (13, 14). At the same time, the health crisis had major consequences on people’s professional lives, and most of them had no choice but to adapt to the new circumstances (15). Many people have been allowed to work remotely but even so, the prevalence of unemployment has increased dramatically, especially among people with different disabilities (16, 17).

The complexity of the relationship between MS and work is noteworthy (6). If it is clear that people without comorbidities suffered direct consequences of the pandemic in their working life, it is reasonable to hypothesize that PwMS, who have always been at risk of unfavorable occupational outcomes, were particularly affected by the pandemic of COVID-19 from the occupational point of view. Moreover, some researchers have already warned about the real risk of further pandemics and categorically stated the importance of scientific research as a tool to improve the management and prevention of other health catastrophes (18). In this scenario, we decided to perform the first systematic review dedicated to exploring the occupational outcomes of PwMS during the COVID-19 pandemic and describe their possible associated factors.

## Methods

### Protocol and registration

The Preferred Reporting Items for Systematic Reviews and Meta-analyses (PRISMA) (19) statement, the Joanna Briggs recommendations for systematic reviews of observational epidemiological studies reporting prevalence and cumulative incidence data (20), and the Meta-analysis of Observational Studies in Epidemiology (MOOSE) indications (21) were followed to conduct this systematic review with meta-analysis. The protocol was registered in the International Prospective Register of Systematic Reviews (PROSPERO) with the registration number CRD42022380463. As this research does not involve the direct recruitment of subjects, the local ethics committee’s approval and the written consent form were not required.

### Data sources and search strategy

A systematic literature search using four electronic academic.

databases—PubMed/MEDLINE, Scopus, SciVerse ScienceDirect, Web of Science — was performed. The following search terms were used: (Employ* OR unemploy* OR occupation* OR “work” OR vocation* OR “workplace” OR “workforce” OR “labour force” OR “labor force” OR Career* OR Job* OR “worker” OR “fitness for work”) AND (“Multiple sclerosis” OR “Demyelinating Autoimmune Diseases” OR “Demyelinating Autoimmune Disorders” OR “Clinically Isolated Syndrome” OR “Demyelinating”) AND (“SARS-CoV-2” OR “COVID-19” OR “Coronavirus disease 2019”). The detailed search strategy is presented in the Supplementary Table S1. The search results were exported and managed in Mendeley 1.19.8 (Elsevier, New York, United States).

### Study selection

Two independent and previously trained investigators (BV and AR) carried out the selection of the studies, one being blind to the decision of the other. In case of conflicting views, a senior investigator (GD) was consulted to promote a discussion and reach a consensus. After removing duplicate entries, we performed a first screening of titles and abstracts to assess their potential relevance and remove those off-topic. Then, the full manuscripts were carefully read to determine their final eligibility. The inclusion criteria were framed according to the PICOS acronym. We included any peer-reviewed original article that enrolled adult patients with the diagnosis of MS and assessed any occupational variable during the COVID-19 pandemic. The studies could have chosen any comparator or intervention. There were no time limits and no language restrictions. We accepted cross-sectional studies, longitudinal studies, and experimental studies, and articles designed as reviews, conference abstracts, letters to the editor, expert opinions, commentaries, case reports, case series, editorials were excluded. We also excluded different published articles that reported the same result from the same study population.

### Outcomes measures

The primary outcomes were the prevalence of unemployment, retirement, and employment status change among people with MS during the COVID-19 pandemic. Other outcomes included the modality and characteristics of work: type of work, full-time work, part-time work, and remote work. We also searched for data from studies that addressed any change in the work status due to the COVID-19 outbreak. The prevalence of all these outcomes was calculated considering the number of events as the numerator and the number of study participants as the denominator. The outcome measures were regarded as categorical variables and reported as percentages.

### Data extraction and quality assessment

The data extracted included information about the first author, country of the study, year of publication, sample size, mean age of participants, higher educational attainment (defined as more than 12 years of schooling), gender distribution, study design, mean duration of the disease, MS phenotype (progressive or relapsing–remitting), Expanded Disability Status Scale (EDSS) scores, history of past COVID-19 infection, use of disease-modifying drugs (DMDs) and the prevalence of anxiety or depression. When available, we recorded a description of which DMD they were using as well as the prevalence of PwMS under high-efficacy therapies (22). We considered as “high efficacy therapy” as the immunotherapies recommended by the current guidelines of treatment of MS that have a greater efficacy: natalizumab, ocrelizumab, rituximab, alemtuzumab, siponimod, cladribine, fingolimod. We considered as “other immunosuppressants” the drugs that are not in the first line of treatment of MS but have a “non-specific” immunosuppressive action: cyclophosphamide, methotrexate, azathioprine, mycophenolate mofetil. This decision is in line with the classification used in the studies included in the review. In the case of articles missing essential data, we contacted the corresponding author to obtain more information by e-mail. The study was excluded whenever our attempt to contact failed. When a multicenter study reported the results according to each country, the information was treated as if it came from two different studies. All extracted data were double-checked 1 month after the initial extraction to optimize intra-rater reliability and minimize the risk of bias. The quality assessment was performed with the Critical Appraisal Checklist for Studies Reporting Prevalence Data which was developed and validated by the Joanna Briggs Institute (23). It comprises 9 questions for which researchers can answer “yes,” “no,” “unclear” or “not applicable (NA)” in response to each item. The greater the number of “no” or “uncertain” selected, the greater the risk of bias in each category and in each study. The critical appraisal was carried out considering the variables of interest in our review. This step was also carried out by two independent and previously trained investigators (BV and AM), being a third researcher (GD) always consulted in case of discrepancy.

### Statistical analysis

All data about any occupational outcome were synthesized narratively according to each study. Quantitative data were pooled in a meta-analysis. Any study that determined as an inclusion criterion the need for some specific occupational characteristic (e.g., subjects necessarily employed) was excluded from the meta-analysis due to the clear addition of a selection bias in the calculation of pooled prevalence. We used the random-effects model based on the binomial distribution to calculate the pooled estimates of the prevalence of unemployment, retirement, and remote working among PwMS with their respective confidence intervals (CIs). Potential influences on prevalence estimates were investigated using subgroup analyses and meta-regression. Therefore, we identified *a priori* potential variables that could be associated with the estimates: age, sex, educational level, disease duration, progressive MS phenotype, EDSS, past COVID-19 infection, DMDs, anxiety, and depression. Potentially statistically significant differences in the effect size of each country were assessed with ANOVA. We assessed the heterogeneity between estimates using the I^2^ statistic and a visual examination of the forest plot. Substantial heterogeneity was considered when I^2^ exceeded 75% (24). To investigate the presence of publication bias, we employed Egger’s linear regression test (25) and examined funnel plots visually. Additionally, we conducted sensitivity analysis, excluding any potential outliers. A *p* < 0.05 was considered statistically significant. All statistical analyses were performed using STATA/BE 17.0.

## Results

**Figure 1:**
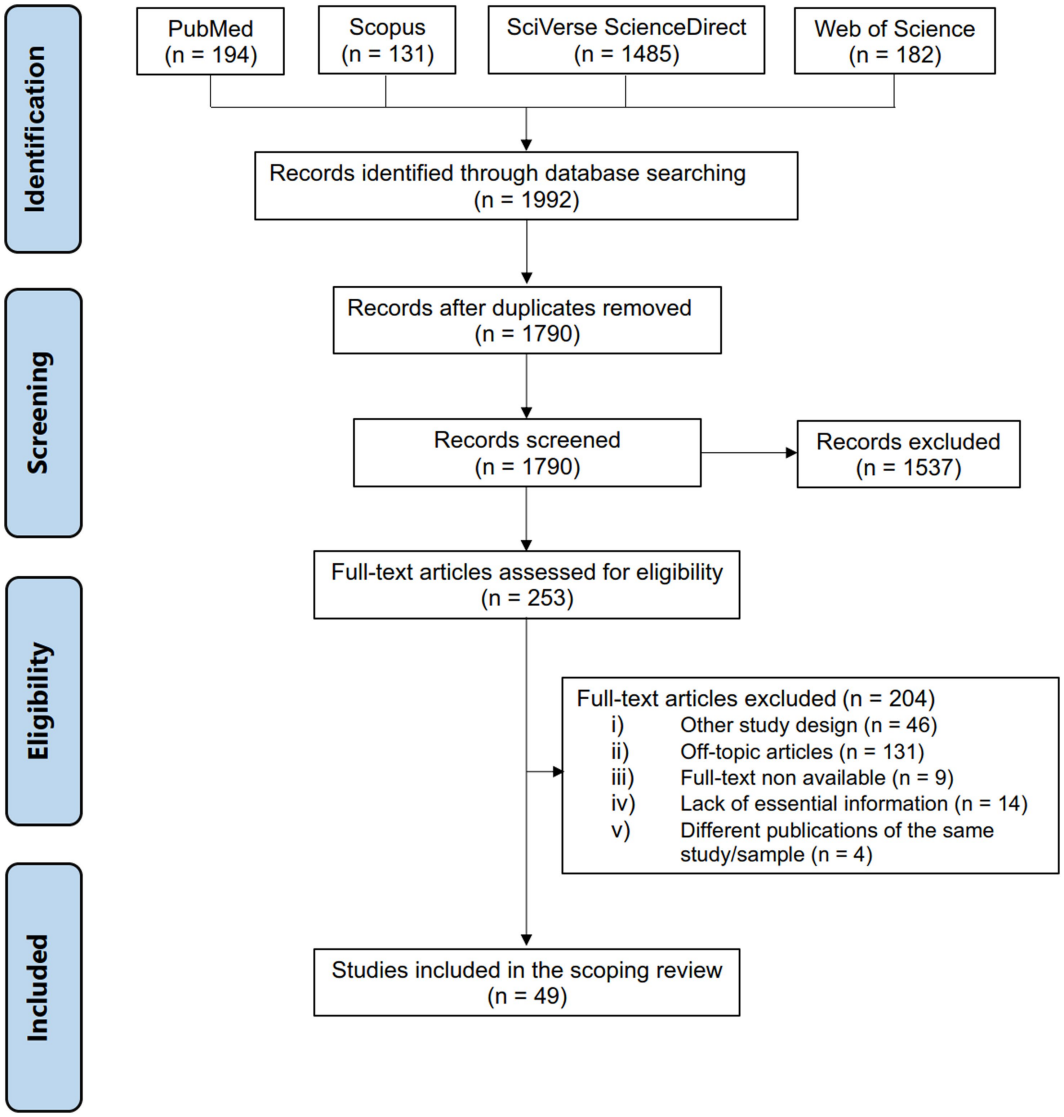
PRISMA flowchart.

One thousand nine hundred and ninety-two articles matched our search terms (Figure 1). After excluding the duplicates, 1790 articles were screened considering the inclusion and exclusion criteria. One thousand seven hundred and forty-one articles did not meet all the eligibility criteria, which led to 49 unique articles being included in our review. The most common reason for exclusion was the study that did not address any of the outcomes of interest to our study (off-topic article). All studies were observational studies, being 45 (91.8%) cross-sectional studies and 4 (8.2%) longitudinal studies (Table 1). Studies were done in Argentina (26), Australia (27–29), Chile (30), China (31, 32), Cuba (32), Egypt (33), Kuwait (33), France (34), Iran (35–40), Italy (41–48), Montenegro (49), Poland (50, 51), Portugal (52), Serbia (53, 54), Spain (31, 32, 55), Turkey (56–58), and the United States of America (47, 59–69). Most of the articles demonstrated to have reasonable methodological quality. Few articles stated that they estimated the sample size before enrolling subjects and only 6 (12.2%) reported the response rate and/or managed it appropriately. The detailed quality assessment is described in the Supplementary Table S2.

**Table 1:** Summary and characteristics of included studies.

**Authors**	**Year**	**Study design**	**Country**	N	Age, mean (SD)	Sex (%)	High educational level (%)	Disease duration, mean (SD)	Progressive MS (%)	EDSS, mean (SD)	COVID-19 infection	USE OF DMD (%)	Unemployed (%)	Unemployed OR Retired (%)	Retirement (%)	**Part-time (%)**	**Full-time (%)**	**Remote work (%)**	**Change in employment (%)**
Zanotto et al.,	2021	cross-sectional	United States of America	106	59.0 (13.0)	78.3	65.1	NA	43.4	NA	NA	NA	4 (3.8)	55 (51.9)	51 (47.9)	7 (6.7)	19 (17.9)	NA	NA
Alschuler et al.,	2021	cross-sectional	United States of America	491	55.8 (12.6)	81.3	89.4	16.7 (11.2)	26.7	NA	0.2	69.9	159 (32.4)	305(62.1)	146 (29.7)	34 (6.9)	144 (29.3)	NA	NA
Pokryszko-Dragan et al.,	2021	cross-sectional	Poland	287	41.0 (72.5)	72.5	54.4	11.8 (NA)	12.2	NA	2.4	81.9	72 (25.1)	150 (52.3)	78 (27.2)	21 (7.3)	147 (51.2)	NA	96 (33.4)
Ehde et al.,	2021	prospective cohort study	United States of America	491	55.7 (12.6)	81.3	71.9	16.7 (11.2)	26.6	NA	3.3	52.5	195 (39.7)	344 (70.1)	149 (30.4)	37 (7.5)	142 (29)	NA	NA
Boulin et al.,	2022	cross-sectional	France	100	46.3 (13.0)	83.0	66.0	NA	NA	NA	0.0	NA	45 (45.0)	45 (45.0)	NA	8 (8.0)	47 (47)	18 (18.0)	NA
Ciampi et al.,	2020	cross-sectional	Chile	400	41.2 (11.4)	71.0	NA	NA	NA	NA	22.0	88.0	41 (10.2)	81 (20.2)	40 (10.0)	44 (11.0)	196 (49)	296 (74.0)	NA
Landi et al.,	2022	cross-sectional	Italy	570	NA	70.0	34.0	NA	NA	NA	NA	84.0	114 (20.0)	217 (38.1)	103 (18.0)	68 (11.9)	242 (42.6)	NA	NA
Lawford et al.,	2022	cross-sectional	Australia	48	NA	NA	NA	NA	NA	NA	NA	NA	31 (65.0)	39 (81.2)	8 (17.0)	7 (15.0)	2 (4.0)	NA	NA
Chen et al.,	2022	cross-sectional	United States of America	70	47.7 (13.0)	81.4	93.1	NA	25.7	NA	26	NA	26 (37.1)	34 (48.6)	8 (11.4)	12 (17.1)	23 (32.9)	NA	NA
Bishop et al.,	2021	cross-sectional	United States of America	69	43.4 (12.7)	75.4	71.1	NA	26.1	NA	NA	88.1	25 (36.2)	25 (36.2)	NA	14 (20.3)	23 (33.3)	NA	33 (47.8)
Zanghì et al.,	2020	cross-sectional	Italy	432	40.4 (12.4)	64.1	NA	5.3 (3.2)	0.0	NA	NA	100.0	NA	NA	NA	NA	NA	NA	NA
Landi et al.,	2020	cross-sectional	Italy	551	44.7 (11.4)	68.0	33.0	NA	NA	NA	NA	88.0	193 (35.0)	193 (35.0)	NA	NA	NA	NA	NA
Naser Moghadasi et al.,	2021	cross-sectional	Iran	133	36.3 (9.2)	94.7	NA	NA	11.3	NA	6.0	81.8	13 (9.8)	13 (9.8)	NA	21 (15.8)	NA	NA	NA
Abbasi et al.,	2022	cross-sectional	Iran	1,479	37.2 (8.8)	79.9	72.5	NA	18.8	NA	28.5	NA	845 (57.1)	845 (57.1)	NA	NA	NA	NA	NA
Ali Sahraian et al.,	2021	cross-sectional	Iran	583	36.2 (8.2)	78.0	74.0	NA	22.0	NA	23	NA	326 (56.0)	326 (55.9)	NA	NA	NA	NA	NA
Alirezaei et al.,	2022	cross-sectional	Iran	282	35.7 (NA)	81.7	73.3	7.4 (NA)	30.09	2.9 (NA)	9.2	NA	175 (62.0)	175 (62.1)	NA	NA	NA	NA	NA
Altunan et al.,	2021	cross-sectional	Turkey	205	37.7 (10.0)	74.1	41.0	30.0 (9.1)	7.9	2.1 (1.4)	NA	81.0	108 (52.7)	108 (57.7)	NA	NA	NA	NA	NA
Arrambide et al.,	2021	retrospective cohort stdy	Spain	326	44.8 (11.5)	67.8	NA	11.0 (8.0)	19.3	NA	100.0	81.9	NA	NA	NA	NA	NA	NA	NA
Bonavita et al.,	2021	cross-sectional	Italy	612	43.0 (10.0)	76.0	30.9	NA	18.6	NA	0.2	89.8	281 (45.9)	329 (53.8)	48 (7.9)	NA	NA	NA	NA
Capuano et al.,	2021	prospective cohort study	Italy	67	37.5 (11.1)	55.2	NA	7.6 (8.1)	NA	NA	7.5	88.1	44 (65.7)	44 (65.7)	NA	NA	NA	16 (23.9)	40 (59.7)
Ciotti et al.,	2022	cross-sectional	United States of America	237	NA	79.3	NA	NA	27.0	NA	14.3	82.7	142 (59.8)	142 (59.9)	NA	NA	NA	NA	NA
Kamel et al.,	2021	retrospective cohort study	Egipt and Kuwait	152	NA	61.8	NA	NA	NA	NA	NA	NA	42 (27.6)	75 (49.3)	33 (21.7)	NA	NA	NA	NA
Krzystanek et al.,	2022	cross-sectional	Poland	248	40.8 (10.6)	77.8	92.7	NA	17.3	NA	19.0	83.5	17 (6.9)	84 (33.9)	67 (27.0)	NA	NA	NA	NA
Lynch et al.,	2022	cross-sectional	United States of America	233	NA	79.0	88.0	NA	19.7	NA	3.4	67.0	NA	NA	NA	NA	NA	NA	103 (44.2)
Moniz Dionisio et al.,	2021	cross-sectional	Portugal	270	NA	76.3	NA	NA	10.7	NA	NA	NA	25 (9.3)	102 (37.8)	77 (28.6)	NA	NA	NA	NA
Morris-Bankole et al.,	2021	cross-sectional	Australia	324	47.7 (12.2)	84.2	NA	NA	24.0	NA	NA	NA	183 (56.5)	183 (56.5)	NA	NA	NA	NA	NA
Moss et al.,	2020	cross-sectional	United States of America and Spain	3,028	50.3 (12.1)	75.0	NA	16.4 (11.1)	27.7	2.8 (2.0)	3.0	77.0	NA	NA	NA	NA	NA	NA	NA
Motolese et al.,	2020	cross-sectional	Italy	60	NA	68.3	31.7	5.1 (5.9)	21.7	NA	0.0	91.7	25 (41.7)	25 (41.7)	NA	NA	NA	34 (56.7)	34 (56.8)
Radulovic et al.,	2020	cross-sectional	Montenegro	101	39.4 (8.7)	75.2	NA	7.0 (5.1)	0.0	NA	NA	100.0	27 (26.7)	27 (26.7)	NA	NA	NA	NA	NA
Ramezani et al.,	2021	cross-sectional	Iran	410	38.6 (10.3)	79.5	45.4	NA	NA	NA	9.3	NA	290 (70.7)	290 (70.7)	NA	NA	NA	NA	NA
Rojas et al.,	2022	cross-sectional	Argentina	275	43.2 (10.9)	59.6	NA	NA	0.0	3.1 (2.0)	NA	NA	65 (23.6)	65 (23.6)	NA	NA	NA	NA	NA
Saeedi et al.,	2021	cross-sectional	Iran	186	34.4 (8.3)	76.9	NA	7.7 (5.3)	29.0	2.7 (1.8)	NA	NA	105 (56.5)	105 (56.4)	NA	NA	NA	NA	NA
Sahraian et al.,	2020	cross-sectional	Iran	233	34.2 (8.0)	77.3	NA	7.4 (5.0)	27.9	NA	NA	NA	98 (42.1)	98 (42.1)	NA	NA	NA	NA	NA
Schwartz et al.,	2022	cross-sectional	Italy	292	44.4 (11.4)	69.0	53.0	NA	NA	NA	10.0	NA	86 (29.0)	NA	16 (5.0)	NA	NA	NA	NA
Schwartz et al.,	2022	cross-sectional	United States of America	416	50.3 (11.0)	85.0	50.0	NA	NA	NA	9.0	NA	203 (48.8)	247 (59.4)	44 (11.0)	NA	NA	NA	NA
Seery et al.,	2020	cross-sectional	Australia	170	40.6 (NA)	77.0	NA	8.5 (8.5)	4.0	2.1 (NA)	NA	NA	NA	NA	NA	NA	NA	63 (37.0)	NA
Sparaco e tal,	2022	cross-sectional	Italy	154	43.8 (10.5)	70.8	NA	16.5 (9.4)	15.6	NA	NA	56.5	14 (9.1)	14 (9.1)	NA	NA	NA	50 (32.4)	NA
Stojanov et al.,	2021	cross-sectional	Serbia	67	45.1 (8.9)	67.8	29.3	NA	0.0	3.6 (1.2)	NA	NA	37 (54.8)	37 (55.2)	NA	NA	NA	NA	NA
Stojanov et al.,	2020	cross-sectional	Serbia	95	43.4 (9.7)	67.6	27.4	NA	0.0	3.6 (1.3)	NA	NA	56 (59.4)	56 (58.9)	NA	NA	NA	NA	NA
Uhr et al.,	2021	cross-sectional	United States of America	610	NA	80.5	NA	14.9 (13.5)	35.1	NA	7.7	70.7	272 (44.8)	373 (61.1)	101 (16.6)	NA	NA	88 (14.5)	NA
Vogel et al.,	2020	cross-sectional	United States of America	1,019	54.2 (NA)	79.0	NA	NA	34.0	NA	0.7	73.0	446 (43.8)	571 (56.0)	125 (12.3)	NA	NA	NA	NA
Xiang et al.,	2021	cross-sectional	United States of America	401	NA	77.8	90.5	NA	20.5	NA	9.3	72.2	219 (54.6)	219 (54.6)	NA	NA	NA	NA	NA
Yalçın et al.,	2021	cross-sectional	Turkey	379	35.8 (9.9)	67.5	70.4	NA	NA	NA	6.9	NA	NA	NA	NA	NA	NA	NA	NA
Yeni et al.,	2022	cross-sectional	Turkey	89	41.1 (10.3)	62.9	36.0	7.7 (6.2)	NA	1.3 (1.6)	NA	NA	7 (7.9)	7 (7.9)	NA	NA	NA	NA	NA
Zhang et al.,	2022	cross-sectional	China	194	33.2 (9.2)	66.0	68.6	NA	NA	NA	NA	NA	NA	NA	NA	NA	NA	NA	51 (26.3)
Zhang et al.,	2021	cross-sectional	China	99	NA	NA	NA	NA	NA	NA	NA	NA	NA	NA	NA	NA	NA	NA	53 (53.6)
Zhang et al.,	2022	cross-sectional	Cuba	104	44.5 (13.6)	80.9	82.5	NA	NA	NA	NA	NA	NA	NA	NA	NA	NA	NA	52 (50.0)
Zhang et al.,	2021	cross-sectional	Spain	153	NA	NA	NA	NA	NA	NA	NA	NA	NA	NA	NA	NA	NA	NA	43 (28.3)
Zhang et al.,	2022	cross-sectional	Spain	63	46.2 (10.2)	70.2	59.6	NA	NA	NA	NA	NA	NA	NA	NA	NA	NA	NA	22 (35.0)

NA = Not available. SD = Standard deviation.

Overall, the total sample size comprised 17,364 individuals with MS. The mean age ranged from 33.2 to 59.0 years and the proportion of women in the studies varied from 55.2 to 94.7%. Between 55.2 and 94.7% of the subjects had a higher educational level. The mean disease duration ranged from 5.1 to 30.0 years and the proportion of subjects diagnosed with the progressive form of MS varied from 0.0 to 43.4%. The prevalence of the use of DMDs ranged between 52.5 to 100.0%.

### Qualitative analysis

Some studies report some associations between employment status and clinical and demographic characteristics. In an American study, PwMS who self-denominated Hispanic, Latinx or Spanish origin had a statistically significantly higher rate of job loss. Moreover, the educational level was also associated with the prevalence of unemployment among PwMS. The proportion of workers who had a university degree and lost their jobs due to COVID-19 was four times lower than that of other workers (14.3% vs. 55.6%, *p* = 0.012) (65). The employment status of PwMS during the COVID-19 pandemic was also associated with the presence of anxious and depressive symptoms (38). In line with this finding, Altunan et al. showed that the use of dysfunctional strategies of coping was statistically higher among the unemployed group of PwMS (56). Regarding the relationship between the occupational outcomes of PwMS and the vaccination against COVID-19, only two studies provided information. Ciotti et al. found that vaccination status was not statistically associated with employment status (66). In contrast, Abbasi et al. reported that being unemployed is associated with COVID-19 vaccine hesitancy/rejection (36).

The results of the present review indicate that a large proportion of PwMS has experienced job changes due to the COVID-19 pandemic. Pokryszko-Dragan et al. reported that 33% of PwMS faced any work-related problem (50). Among these, 41.7% had their job suspended, 14.6% were fired, 14.6% had some difficulties in turning to remote work, 8.3% had an increased workload and 20.8% feared the infection at the workplace. In line with these findings, Bishop et al. demonstrated that, among participants previously engaged in full-time employment before the onset of the COVID-19 pandemic, 17.4% reported job loss due to the pandemic, while 39.1% reported other alterations in their employment status (65). The most commonly reported changes encompassed a reduction in working hours or shifts, transitioning to remote work, and switching departments or job roles within their respective organizations. Capuano et al. found that only 10.5% of the research subjects continued to work in the same modality as before the lockdown, while 23.9% started working from home (45). Motolese et al. also described that 26.3% had a working hour reduction and only 43.2% kept the usual workplace (46). In a different scenario, an American cross-sectional study revealed that more than half (55.8%) of the PwMS did not have their jobs affected by the pandemic (67). The other participants who had employment somehow changed included 22.7% who reported increased remote work, 11.2% who reported working more than usual, and 11.2% who had to work with children in the house. Zanghì et al. showed that their study participants reported a reduction in the working hours with the lockdown, however, the average pre-pandemic working hours went from 41.3 (SD 4.2) hours per week to an average of 36.2 (SD 3.2) hours per week, indicating that the reduction may not be so pronounced (the authors did not address this data with a statistical test) (42).

There were studies in which no subjects had COVID-19 and others in which the entire sample was diagnosed with the infection. Few studies sought to classify the entire sample according to the type of work performed. In a Polish study, among those who were working at the time of the study, 82.2% were white-collar workers and 17.8% were blue-collar workers (51). In an Italian study, 77.5% of the sample were employees or self-employed, while 22.5% were executives with management roles (42). Arrambide et al. found that healthcare workers with MS had a significantly higher incidence of death from COVID-19 than other PwMS (55).

### Quantitative analysis

**Figure 2:**
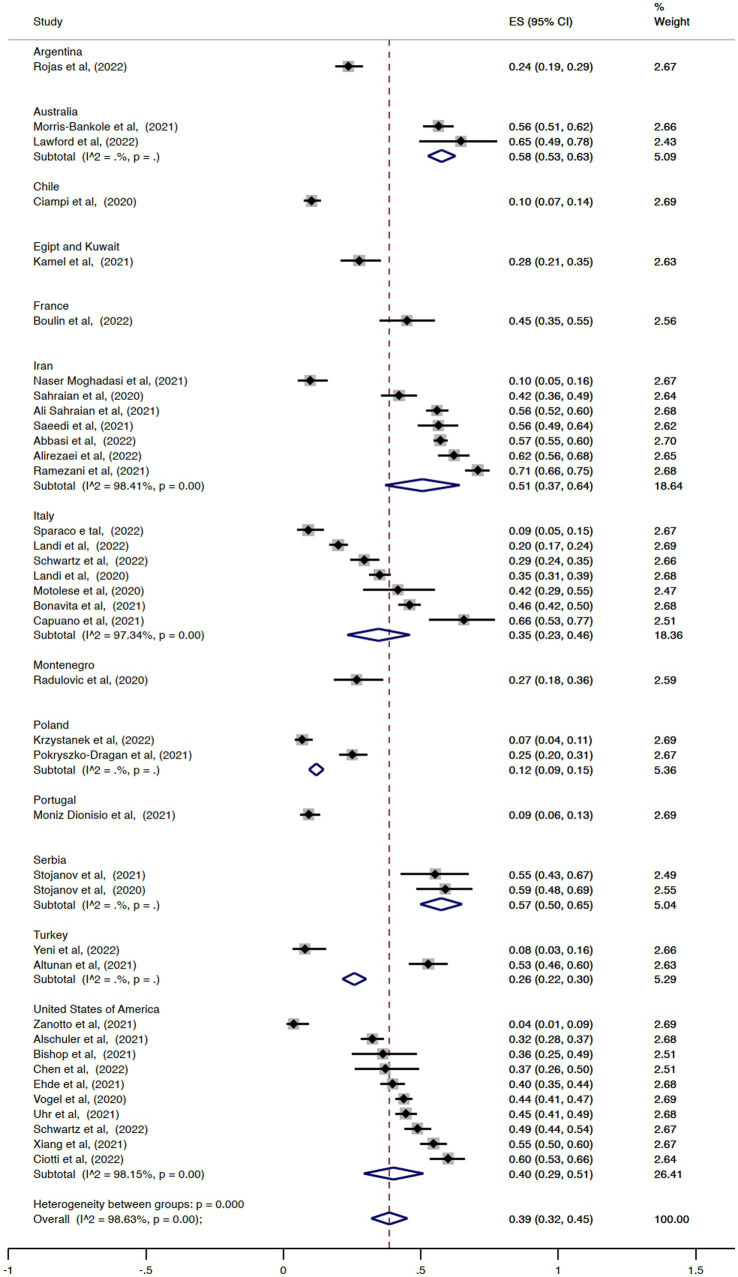
Forest plot with the results of the meta-analysis of unemployment.

**Figure 3:**
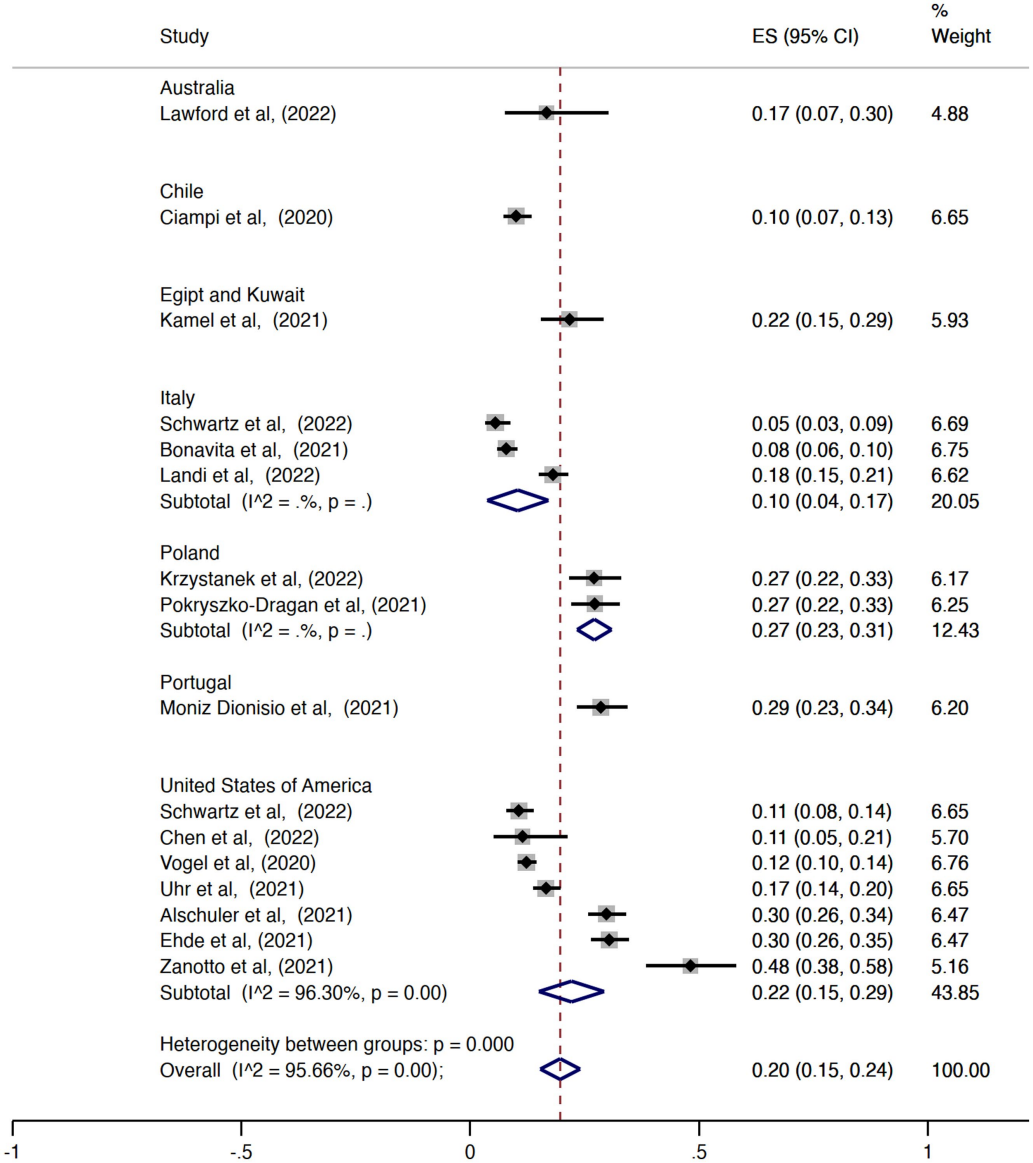
Forest plot with the results of the meta-analysis of retirement.

**Figure 4:**
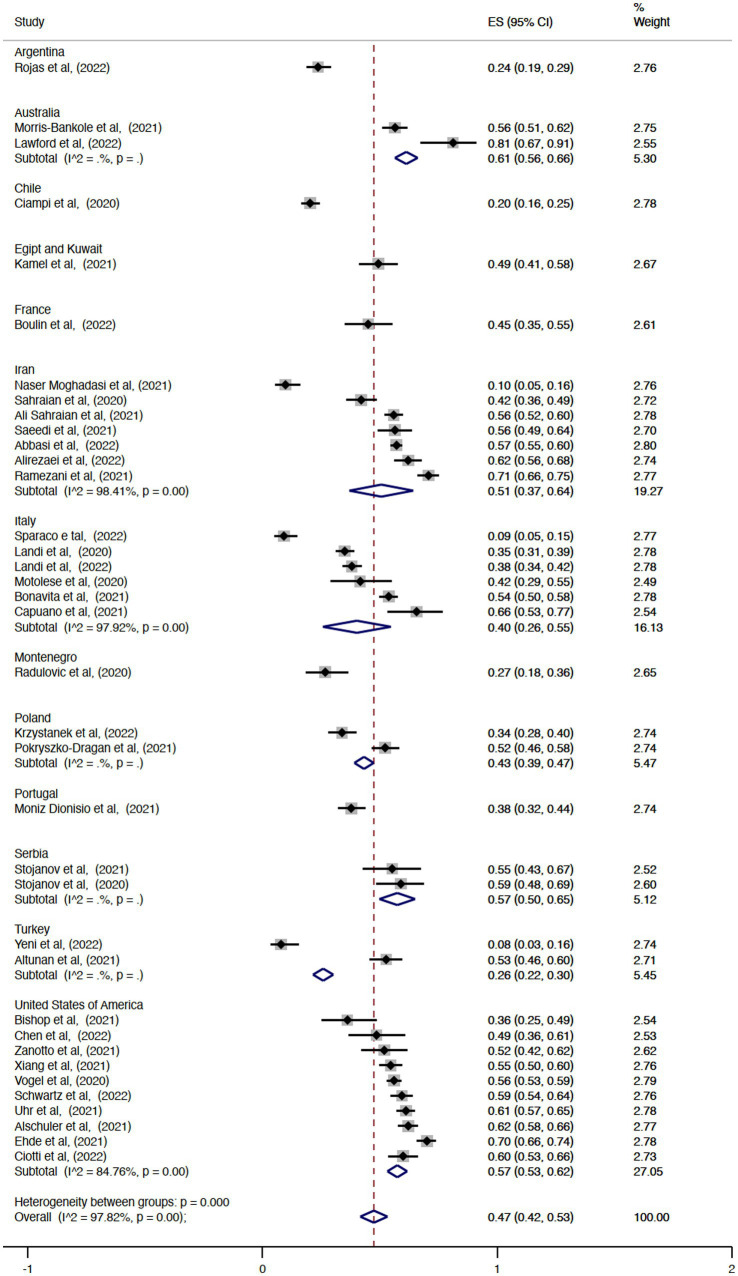
Forest plot with the results of the meta-analysis of unemployment and retirement.

The pooled overall effect size for the prevalence of unemployment was 0.39 (95% CI = 0.32–0.45, I^2^ = 98.63%; Figure 2). There was no statistically significant difference between the pooled estimates of unemployment among the countries (*p* = 0.14). The country with the highest prevalence was Australia (ES = 0.58, 95% CI = 0.53–0.63), and the country with the lowest effect size was Chile (ES = 0.10, 95% CI = 0.07–0.14). Regarding the prevalence of retirement, the pooled overall effect size was 0.20 (95% CI = 0.15–0.24, I^2^ = 95.66%; Figure 3). The effect sizes also did not vary significantly by country (*p* = 0.62), with Italy and Chile having the lowest values (ES = 0.10, 95% CI = 0.04–0.17 and ES = 0.10, 95% CI = 0.07–0.13, respectively) and Portugal the highest (ES = 0.29, 95% CI = 0.23–0.34). Considering the proportion of subjects with MS either unemployed or retired, the pooled overall effect size was 0.47 (95% CI = 0.42–0.53, I^2^ = 97.82%; Figure 4).

**Figure 5:**
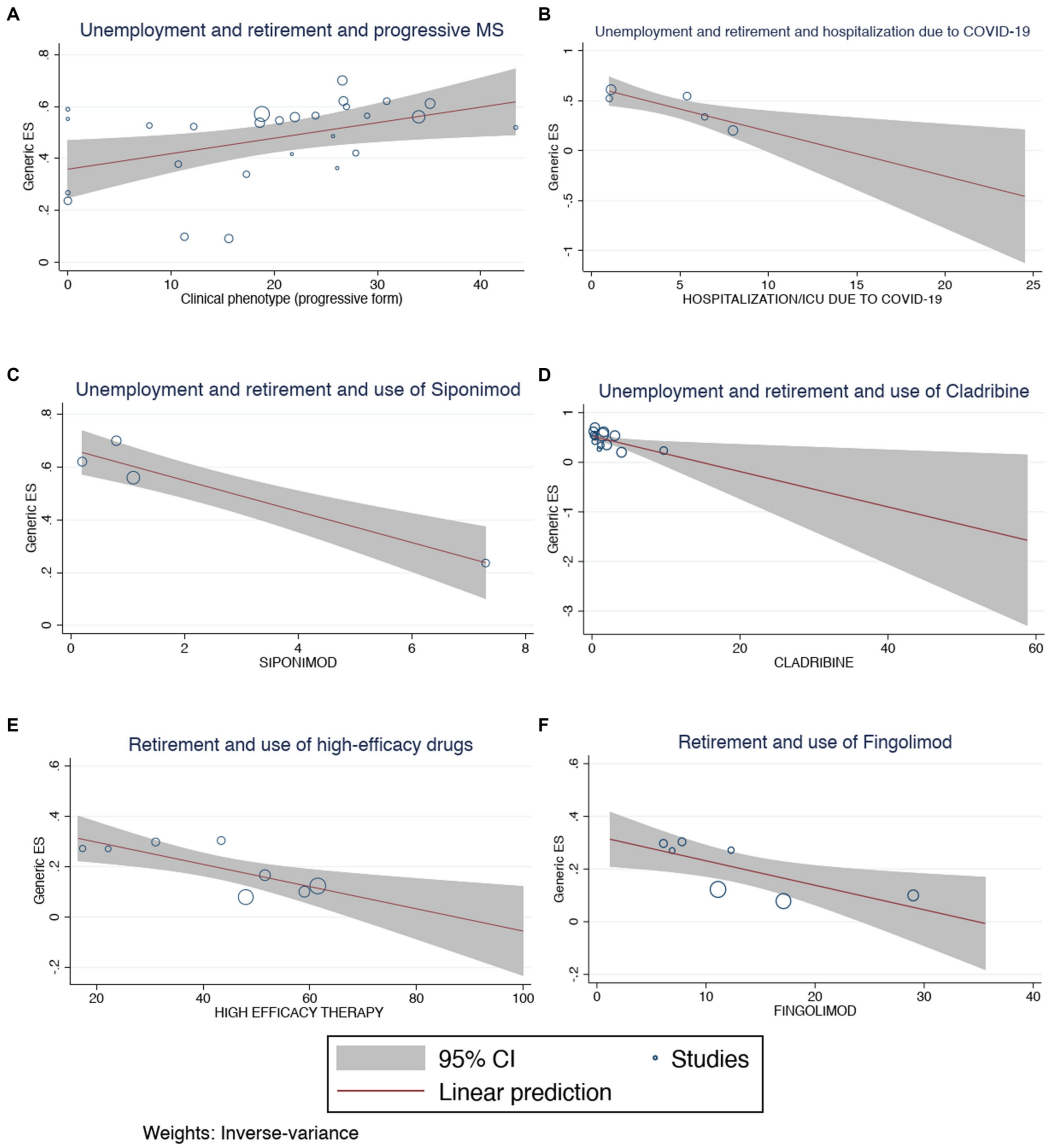
Meta-regression analysis for **(A)** unemployment and retirement and hospitalization due to COVID-19, **(B)** unemployment and retirement and use of cladribine, **(C)** unemployment and retirement and use of siponimod, **(D)** unemployment and retirement and progressive MS, **(E)** retirement and use of fingolimod, **(F)** retirement and use of high-efficacy drugs.

On meta-regression analysis, unemployment was negatively associated with the use of other immunosuppressants (*p* = 0.032; Supplementary Figure S1). The pooled prevalence of PwMS who were unemployed or retired was positively associated with the progressive phenotype of the disease (*p* = 0.017; Figure 5A) and the use of glatiramer acetate (*p* = 0.004; Supplementary Figure S2), but negatively associated with hospitalization due to COVID-19 (*p* = 0.008; Figure 5B) and the use of other immunosuppressants (*p* = 0.032; Supplementary Figure S3), siponimod (*p* < 0.001; Figure 5C), and cladribine (*p* = 0.021; Figure 5D). In parallel, the estimate of the prevalence of retirement was negatively associated with the use of high-efficacy therapies (*p* = 0.004; Figure 5E), fingolimod (*p* = 0.014; Figure 5F), cladribine (*p* < 0.001; Supplementary Figure S4) and the presence of depressive symptoms (*p* < 0.001; Supplementary Figure S5). The inspection of the funnel plot and the results of the Egger’s test showed a publication bias in the pooled estimates of unemployment (*p* = 0.02; Supplementary Figure S6) and retirement (*p* = 0.014; Supplementary Figure S7) but not in the estimate of PwMS who were either unemployed or retired (*p* = 0.509; Supplementary Figure S8). The sensitivity analysis did not show any other statistically significant results.

**Figure 6:**
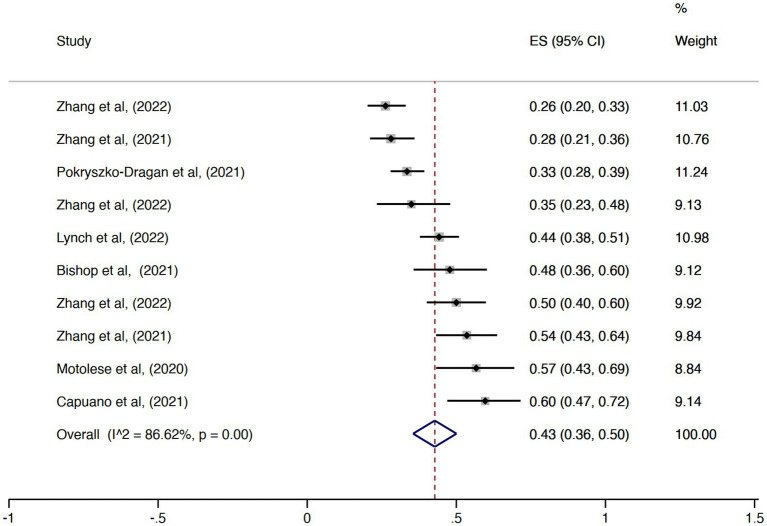
Forest plot with the results of the meta-analysis of change in the employment status.

**Figure 7:**
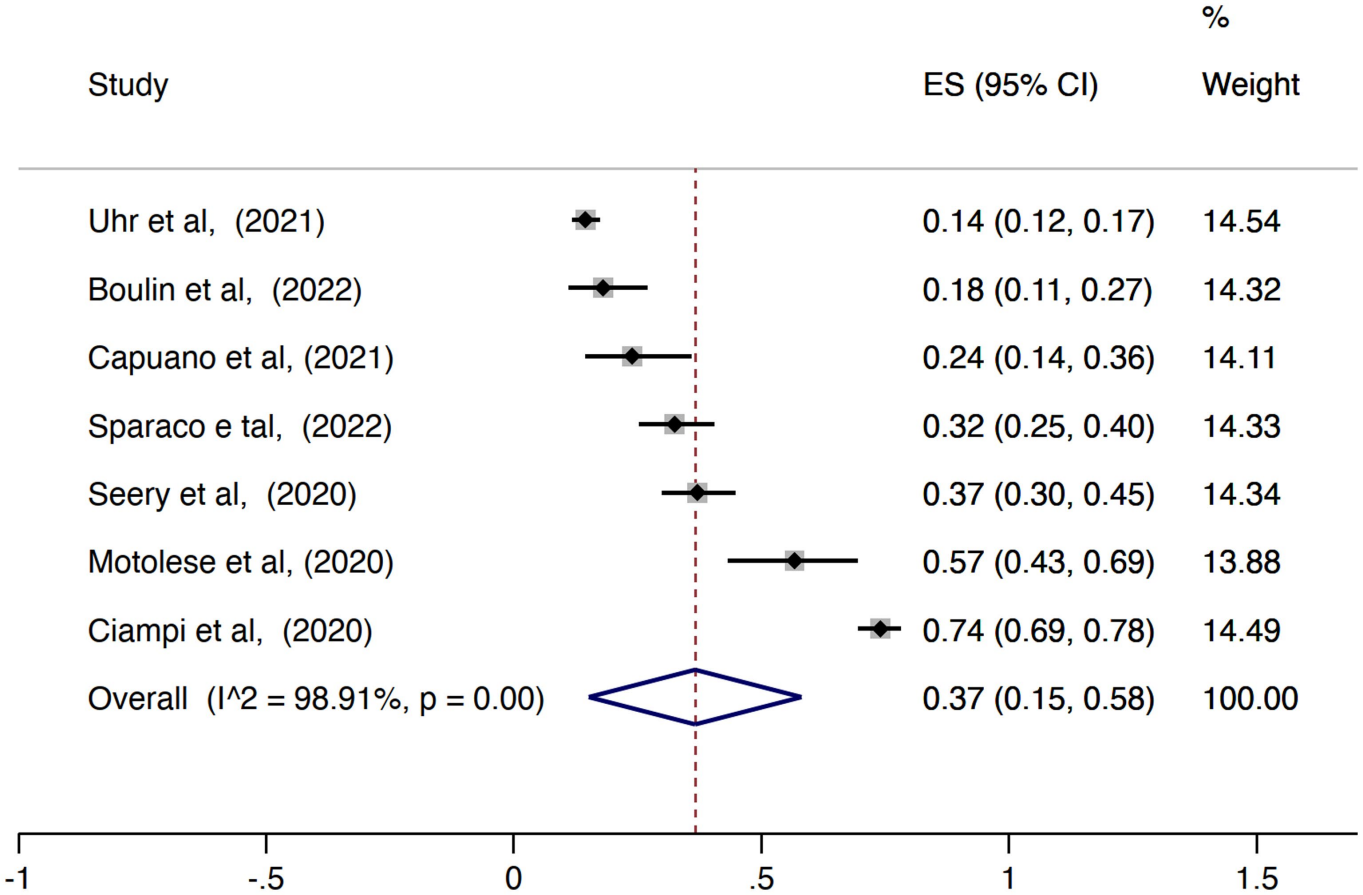
Forest plot with the results of the meta-analysis of remote work.

**Figure 8:**
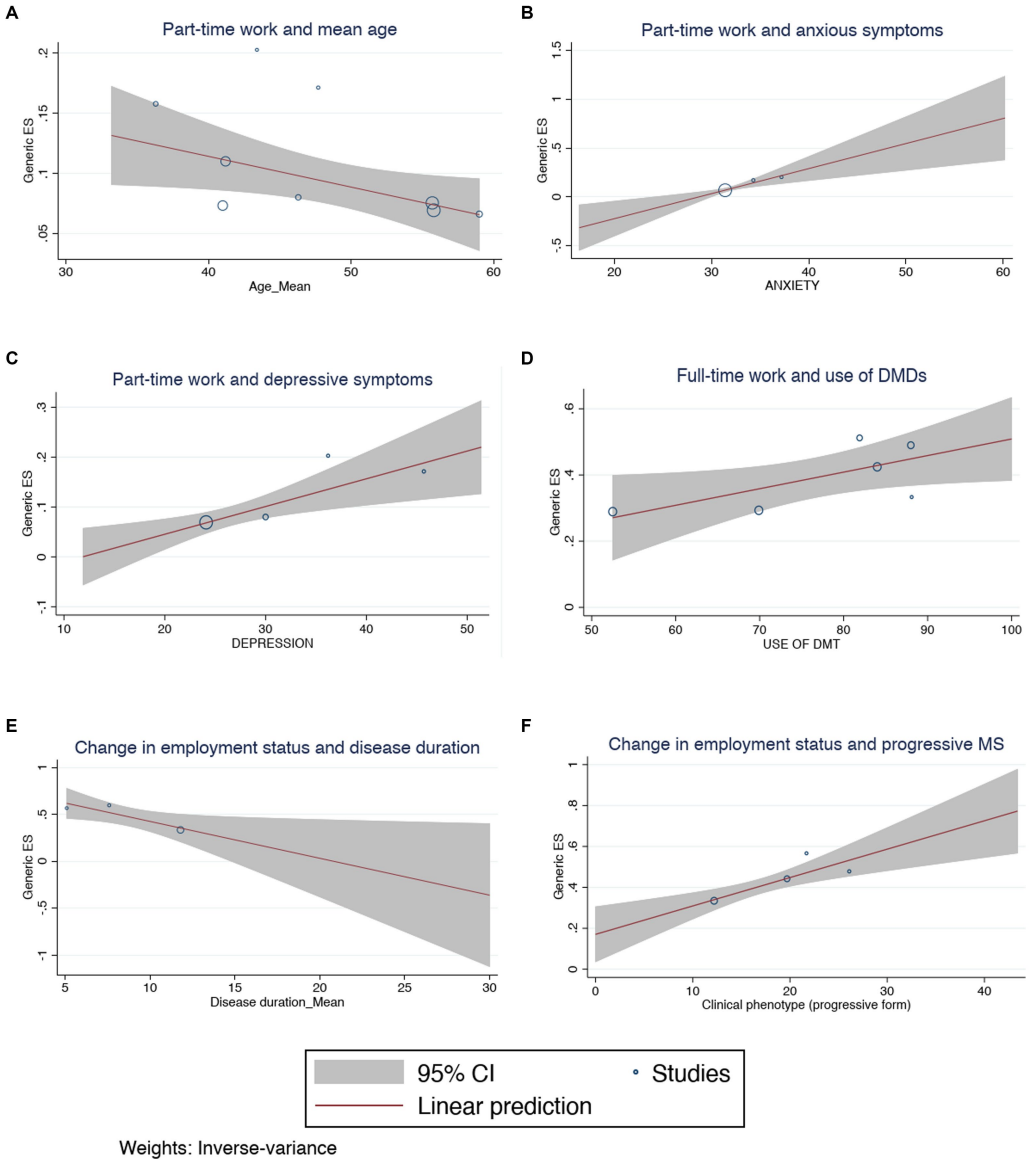
Meta-regression analysis for **(A)** change in employment status and disease duration, **(B)** change in employment status and progressive MS, **(C)** part-time work and anxious symptoms, **(D)** part-time work and mean age, **(E)** part-time work and depressive symptoms, **(F)** full-time work and use of DMDs.

The pooled proportion of PwMS that reported any change of the employment status during the COVID-19 pandemic was 0.43 (95% CI = 0.36–0.50, I^2^ = 86.62%; Figure 6) while the pooled prevalence of PwMS who worked remotely during this period was 0.37 (95% CI = 0.15–0.58, I2 = 98.91%; Figure 7). There was no statistically significant difference considering the study origin for the combined estimates of change in employment status (*p* = 0.30) and remote working (*p* = 0.37). The change in employment status was negatively associated with the duration of MS (*p* = 0.03; Figure 8A) but positively associated with the progressive phenotype of the disease (*p* < 0.001; Figure 8B). There were no statistically significant results in the meta-regression analyses for the proportion of PwMS working remotely.

Regarding the workload of the subjects included in the studies, the estimates of the effect sizes of the people working part-time and full-time were 0.10 (95% CI = 0.08–0.12, I^2^ = 64.15%; Supplementary Figure S9) and 0.34 (95% CI = 0.25–0.43, I^2^ = 96.1%; Supplementary Figure S10). The pooled prevalence of PwMS who were working part-time was positively influenced by the older average age of the individuals (*p* = 0.034; Figure 8C), the diagnosis of COVID-19 (*p* = 0.005; Supplementary Figure S11), the use of fingolimod (*p* = 0.032; Supplementary Figure S12), alemtuzumab (*p* = 0.04; Supplementary Figure S13) and cladribine (*p* = 0.02; Supplementary Figure S14), and the presence of anxious (*p* = 0.001; Figure 8D) and depressive symptoms (*p* = 0.003; Figure 8E). In contrast, the prevalence of PwMS who had a full-time job was associated with younger mean age (*p* < 0.001; Supplementary Figure S15), a shorter disease duration (*p* < 0.001; Supplementary Figure S16), the progressive form of MS (*p* < 0.001; Supplementary Figure S17), the use of any DMD (*p* = 0.038; Figure 8F), and the use of glatiramer acetate (*p* = 0.001; Supplementary Figure S18), alemtuzumab (*p* = 0.035; Supplementary Figure S19) and natalizumab (*p* = 0.011; Supplementary Figure S20). There were publication biases in the results of the meta-analysis of the estimates of PwMS working part-time (*p* = 0.005; Supplementary Figure S21), full-time (*p* = 0.005; Supplementary Figure S22) and that had a change in the employment status (*p* = 0.0059; Supplementary Figure S23).

## Discussion

To the best of our knowledge, this is the first systematic review with meta-analysis addressing the occupational outcomes of patients with a neurological disorder during the COVID-19 pandemic. The review provides the first evidence of the occupational impact of the pandemic on PwMS. About half PwMS was either retired or unemployed during the COVID-19 global health crisis. The proportion of PwMS not working in this period is much higher than the prevalence reported in several previous observational studies that investigated unemployment among people from socially disadvantaged areas, people with psychiatric illnesses, health care workers, and the general population (70–73). In addition, this proportion is also higher than the global proportion indicated by the International Labour Organization, which states that only 0.9% of all lost working hours in the pandemic were explained by unemployment and that the unemployment rate increased by 1.1 percentage points to 6.5% in 2020 (74). Undoubtedly, this finding confirms the vulnerability of this special group of patients to unfavorable occupational outcomes. Nevertheless, this finding is comparable to the estimates of the prevalence of unemployment and early retirement in the 40 years before the outbreak (11). This observation may be explained by the implementation and effectiveness of reasonable job accommodations for PwMS. The scientific literature has already described the variety of possibilities of job accommodations that can be applied to the reality of workers with MS to avoid adverse occupational outcomes (10). Moreover, laws, public policies, and collective agreements stimulated by the authorities may have contributed to preventing the definitive withdrawal of disabled workers from the labor force during this period (75, 76).

In general, clinical features associated with higher disease severity were associated with a higher prevalence of unemployment and/or early retirement during the COVID-19 pandemic. The progressive form of the disease and a diagnosis of depression have already been associated with a higher risk of negative occupational outcomes (77–79). The use of other immunosuppressants and more recent and highly effective DMDs have also been related to this type of outcome. The first case may be explained by the fact that immunosuppressants are not the first line of treatment for MS according to the current guidelines and their use may still occur in centers that do not have easy access to highly effective DMDs and in cases of suboptimal responders, who are intolerant or experience adverse reactions to disease-modifying treatments, or for patients who exhibit an aggressive disease course (80). In the second case, the use of highly effective DMDs may be explained by a more severe disease as well, but also by a delay in initiation of high efficacy therapies due to the well-known unequal access to treatment across MS centers and countries (81, 82). Curiously, being hospitalized with the SARS-CoV-2 infection was negatively associated with the pooled prevalence of unemployment and retirement. This observation may be explained by the fact that the workplace represents a risk factor for COVID-19 infection (83), which can naturally increase the risk of hospitalization.

A significant number of PwMS reported changes in their employment status and working remotely during the pandemic. The number is superior to the prevalence reported in most previous studies, indicating that PwMS may be particularly sensitive to job changes (84–87). Furthermore, the changes in the employment status of PwMS are even higher than those experienced by people with disabilities in general. Houtenville et al. showed that the percentage of employed people with disabilities dropped from 31.1 to 26.4% (a relative reduction of 15.1%) (87). PwMS with progressive disease phenotype and longer duration of disease were more at risk of any change in employment status, confirming that the degree of disability and the severity of MS may have left patients at a greater disadvantage from an employment perspective. Working remotely was a strategy well disseminated among PwMS during the pandemic. In general, the possibility of teleworking was particularly widespread among workers with vulnerabilities and underlying medical conditions to address the recommendations of social distancing and work safety for groups at a higher risk for severe COVID-19 (88). Most people were working full-time, suggesting that workload reduction may have been less important in managing the crisis at work. Even though, the diagnosis of more vulnerable conditions among PwMS may have guided employers and/or employees to migrate to part-time work as individuals using DMDs known to cause greater immunosuppression and usually indicated in cases of more severe disease were positively associated with the prevalence of part-time work.

The qualitative analysis from individual studies describes that a high proportion of PwMS described some kind of change at work and that losing their job was not the only possible occupational outcome during the COVID-19 pandemic. Moreover, socially vulnerable groups of PwMS such as ethnic minorities and low-skilled workers may have been more affected by the crisis from an occupational point of view. There were conflicting results on the effectiveness of COVID-19 vaccination in preventing unfavorable occupational outcomes in PwMS. Nevertheless, most of the studies included in the review were conducted before vaccines were available or concurrently with the initial phase of vaccination campaigns around the world, and, therefore, future studies may clarify this issue. Unfortunately, there is still limited evidence on some issues. For instance, PwMS with jobs with different degrees of exposure to COVID-19 were not compared separately. Aspects related to the return to work and job satisfaction were less explored. It is also noteworthy that disability status (assessed with the EDSS) was a less investigated variable in the studies included in the review. No study evaluated possible confounders possibly associated with occupational variables, such as the need for personal reorganization due to the circumstances caused by the pandemic, such as school closures. Moreover, the articles did not report the details of the level of restrictions and national policies implemented during the pandemic and, therefore, no analysis was carried out investigating this aspect with occupational outcomes. The role of the occupational physician and the efficacy of job accommodations provided during the pandemic was not investigated as well.

Our study has several limitations that warrant acknowledgment to ensure a precise interpretation of the results. While the variables under consideration are relatively straightforward to measure, the aggregation of diverse study types introduces the possibility of variations in outcome assessment methods, potentially leading to methodological bias and substantial heterogeneity. Given that the majority of studies employed a cross-sectional design, it remains challenging to establish definitive causal relationships between the occupational consequences for people with multiple sclerosis (PwMS) and the COVID-19 pandemic. Furthermore, there was an uneven distribution of available literature across different countries, potentially limiting the generalizability of our findings to specific regions or nations.

## Conclusion

PwMS are vulnerable to unfavorable occupational outcomes, especially in global catastrophes. The pandemic of COVID-19 challenged health systems and subjected PwMS to inevitable changes in the way they work. Our seminal review can serve as an example of how patients with neurological diseases or disabilities, in general, may have their jobs impacted in a pandemic and foster the context of a global socio-economic crisis. Furthermore, we demonstrated that DMDs can play a crucial role also impacting the occupational outcomes of PwMS. Finally, the results can guide public authorities and physicians to make more effective decisions aimed at optimizing the occupational outcomes of workers with MS even in challenging situations.

## Data availability statement

The original contributions presented in the study are included in the article/Supplementary material, further inquiries can be directed to the corresponding author.

## Author contributions

BV, AR, AM, GD, and PD conceived the study, developed the protocol, and participated in the data interpretation and comments on the paper. BV, AR, and AM performed the search strategy, screened the search results, extracted the data, and performed the data analysis. BV wrote the original manuscript. PD was responsible for coordinating the research. All authors reviewed the manuscript, contributed to the article, and approved the submitted version.
