# Supplementary material for: Occupational outcomes of people with multiple sclerosis during the COVID-19 pandemic: a systematic review with meta-analysis
Source: Front Public Health. 2023 Nov 27;11:1217843. doi: 10.3389/fpubh.2023.1217843 (PMC10711111; doi:10.3389/fpubh.2023.1217843)
Supplement: Supplementary file 1 [file Data_Sheet_1.docx]

**Supplementary material**

Table S1. Detailed search strategy.

| **PubMed** | (Employ* OR unemploy* OR occupation* OR “work” OR vocation* OR “workplace” OR “workforce” OR “labour force” OR “labor force” OR Career* OR Job* OR “worker” OR “fitness for work”) AND (“Multiple sclerosis” OR “Demyelinating Autoimmune Diseases” OR “Demyelinating Autoimmune Disorders” OR “Clinically Isolated Syndrome” OR “Demyelinating”) AND (“SARS-CoV-2” OR “COVID-19” OR “Coronavirus disease 2019”) |
| --- | --- |
| **Scopus** | TITLE-ABS KEY [(Employ* OR unemploy* OR occupation* OR “work” OR vocation* OR “workplace” OR “workforce” OR “labour force” OR “labor force” OR Career* OR Job* OR OR “worker” OR “fitness for work”) AND (“Multiple sclerosis” OR “Demyelinating Autoimmune Diseases” OR “Demyelinating Autoimmune Disorders” OR “Clinically Isolated Syndrome” OR “Demyelinating”) AND (“SARS-CoV-2” OR “COVID-19” OR “Coronavirus disease 2019”)] |
| **SciVerse Science Direct** | ("employ" OR "occupation" OR "work” OR "job") AND (“Multiple sclerosis” OR “demyelinating”) AND (“SARS-CoV-2” OR “COVID-19”) |
| **Web of Science** | (Employ* OR unemploy* OR occupation* OR “work” OR vocation* OR “workplace” OR “workforce” OR “labour force” OR “labor force” OR Career* OR Job* OR “worker” OR “fitness for work”) AND (“Multiple sclerosis” OR “Demyelinating Autoimmune Diseases” OR “Demyelinating Autoimmune Disorders” OR “Clinically Isolated Syndrome” OR “Demyelinating”) AND (“SARS-CoV-2” OR “COVID-19” OR “Coronavirus disease 2019”) |

Table S2. Quality assessment of the articles included int the study.

| **Authors** | **Q1** | **Q2** | **Q3** | **Q4** | **Q5** | **Q6** | **Q7** | **Q8** | **Q9** |
| --- | --- | --- | --- | --- | --- | --- | --- | --- | --- |
| Abbasi et al. | YES | NO | UNCLEAR | YES | YES | UNCLEAR | YES | YES | UNCLEAR |
| Ali Sahraian et al. | YES | NO | UNCLEAR | YES | YES | UNCLEAR | YES | YES | UNCLEAR |
| Alirezaei et al. | YES | YES | UNCLEAR | YES | YES | UNCLEAR | YES | YES | NO |
| Alschuler et al. | YES | NO | YES | YES | YES | YES | YES | YES | YES |
| Altunan et al. | YES | UNCLEAR | UNCLEAR | YES | YES | UNCLEAR | YES | YES | NO |
| Arrambide et al. | YES | UNCLEAR | UNCLEAR | YES | YES | YES | YES | YES | NO |
| Bishop et al. | YES | NO | UNCLEAR | YES | YES | YES | YES | YES | NO |
| Bonavita et al. | YES | NO | YES | YES | YES | YES | YES | YES | NO |
| Boulin et al. | NO | NO | UNCLEAR | YES | YES | YES | YES | YES | NO |
| Capuano et al. | NO | NO | UNCLEAR | YES | YES | UNCLEAR | YES | YES | YES |
| Chen et al. | YES | NO | UNCLEAR | YES | YES | YES | YES | YES | NO |
| Ciampi et al. | YES | UNCLEAR | UNCLEAR | YES | YES | YES | YES | YES | NO |
| Ciotti et al. | YES | YES | UNCLEAR | YES | YES | NO | YES | YES | NO |
| Ehde et al. | YES | YES | UNCLEAR | YES | YES | YES | YES | YES | YES |
| Hollen et al. | YES | YES | UNCLEAR | YES | YES | UNCLEAR | YES | YES | YES |
| Kamel et al. | YES | YES | UNCLEAR | YES | YES | YES | YES | YES | NO |
| Krzystanek et al. | YES | YES | UNCLEAR | YES | YES | YES | YES | YES | NO |
| Landi et al. | YES | YES | UNCLEAR | YES | YES | YES | YES | YES | NO |
| Landi et al. | YES | YES | UNCLEAR | YES | UNCLEAR | UNCLEAR | YES | YES | NO |
| Lawford et al. | YES | YES | YES | YES | YES | YES | YES | YES | NO |
| Lynch et al. | YES | UNCLEAR | UNCLEAR | YES | YES | NO | YES | YES | NO |
| Moniz Dionisio et al. | YES | YES | NO | YES | YES | YES | YES | YES | NO |
| Morris-Bankole et al. | YES | YES | NO | YES | YES | UNCLEAR | YES | YES | NO |
| Moss et al. | YES | YES | UNCLEAR | YES | YES | YES | YES | YES | YES |
| Motolese et al. | YES | YES | YES | YES | YES | UNCLEAR | YES | YES | NO |
| Naser Moghadasi et al. | YES | YES | UNCLEAR | YES | UNCLEAR | YES | YES | YES | NO |
| Pokryszko-Dragan et al. | YES | NO | UNCLEAR | YES | YES | YES | YES | YES | NO |
| Radulovic et al. | YES | UNCLEAR | UNCLEAR | YES | YES | UNCLEAR | YES | YES | NO |
| Ramezani et al. | YES | UNCLEAR | UNCLEAR | YES | YES | UNCLEAR | YES | YES | NO |
| Rojas et al. | YES | YES | YES | YES | YES | UNCLEAR | YES | YES | NO |
| Saeedi et al. | YES | UNCLEAR | UNCLEAR | YES | YES | UNCLEAR | YES | YES | NO |
| Sahraian et al. | YES | UNCLEAR | NO | YES | YES | UNCLEAR | YES | YES | NO |
| Schwartz et al. | YES | YES | NO | YES | YES | YES | YES | YES | NO |
| Schwartz et al. | YES | YES | NO | YES | YES | YES | YES | YES | NO |
| Seery et al. | YES | YES | UNCLEAR | YES | YES | YES | YES | YES | NO |
| Sparaco e tal. | YES | UNCLEAR | UNCLEAR | YES | YES | UNCLEAR | YES | YES | YES |
| Stojanov et al. | YES | UNCLEAR | UNCLEAR | YES | YES | UNCLEAR | YES | YES | NO |
| Stojanov et al. | YES | UNCLEAR | UNCLEAR | YES | YES | UNCLEAR | YES | YES | NO |
| Uhr et al. | YES | UNCLEAR | UNCLEAR | YES | UNCLEAR | YES | YES | YES | NO |
| Vogel et al. | YES | UNCLEAR | UNCLEAR | YES | YES | YES | YES | YES | NO |
| Xiang et al. | YES | YES | YES | YES | YES | UNCLEAR | YES | YES | NO |
| Yalçın et al. | YES | YES | UNCLEAR | YES | YES | NO | YES | YES | NO |
| Yeni et al. | YES | YES | UNCLEAR | YES | YES | YES | YES | YES | NO |
| Zanghì et al. | YES | UNCLEAR | UNCLEAR | YES | YES | YES | YES | YES | NO |
| Zanotto et al. | YES | UNCLEAR | UNCLEAR | YES | UNCLEAR | YES | YES | YES | NO |
| Zhang et al. | YES | UNCLEAR | UNCLEAR | YES | YES | UNCLEAR | YES | YES | NO |
| Zhang et al. | YES | UNCLEAR | UNCLEAR | YES | YES | UNCLEAR | YES | YES | NO |
| Zhang et al. | YES | UNCLEAR | UNCLEAR | YES | YES | UNCLEAR | YES | YES | NO |
| Zhang et al. | YES | NO | UNCLEAR | NO | YES | UNCLEAR | YES | YES | NO |
| Zhang et al. | YES | NO | UNCLEAR | NO | YES | UNCLEAR | YES | YES | NO |
