# Supplementary figures and images for: Occupational outcomes of people with multiple sclerosis during the COVID-19 pandemic: a systematic review with meta-analysis
Source: Front Public Health. 2023 Nov 27;11:1217843. doi: 10.3389/fpubh.2023.1217843 (PMC10711111; doi:10.3389/fpubh.2023.1217843)

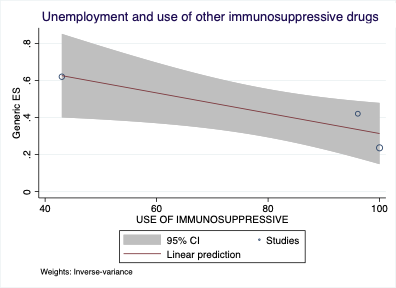

Supplement: Supplementary file 2 [file Image_1.TIFF]

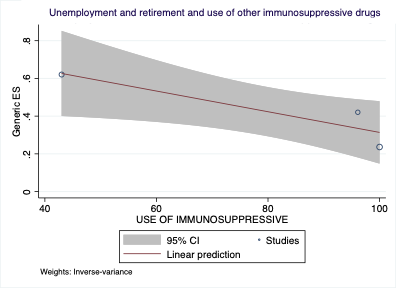

Supplement: Supplementary file 3 [file Image_2.TIFF]

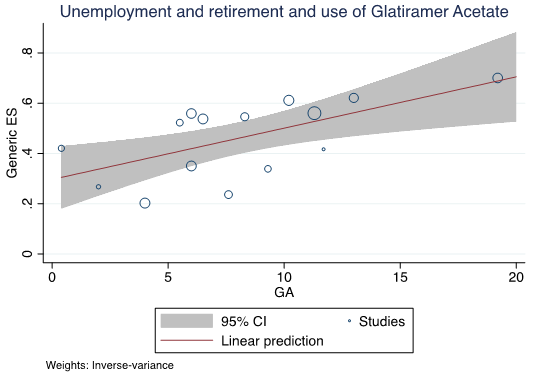

Supplement: Supplementary file 4 [file Image_3.TIF]

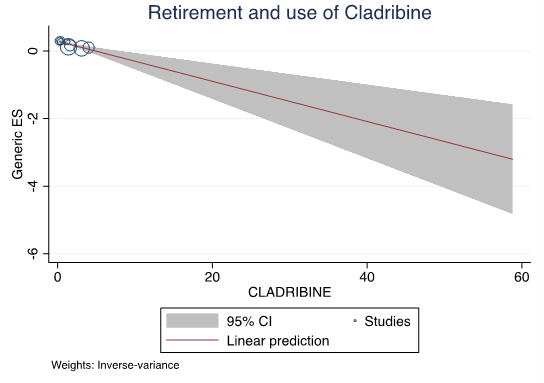

Supplement: Supplementary file 5 [file Image_4.TIF]

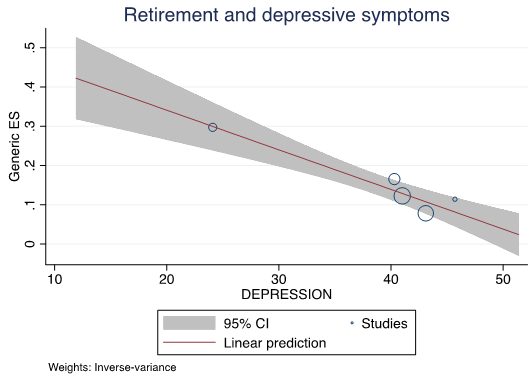

Supplement: Supplementary file 6 [file Image_5.TIF]

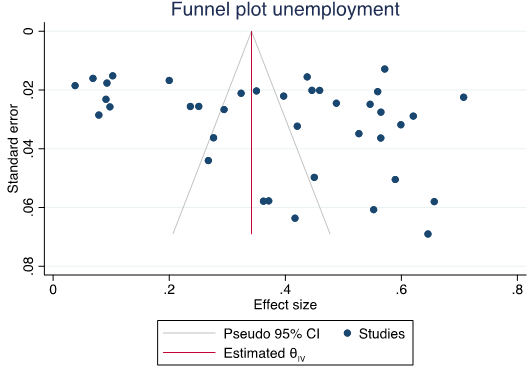

Supplement: Supplementary file 7 [file Image_6.TIF]

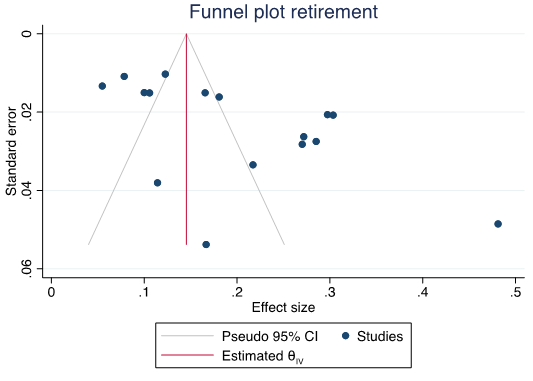

Supplement: Supplementary file 8 [file Image_7.TIF]

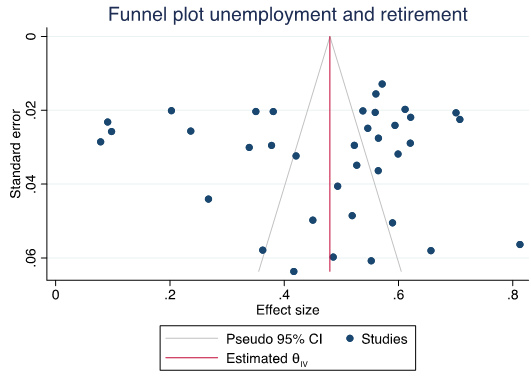

Supplement: Supplementary file 9 [file Image_8.TIF]

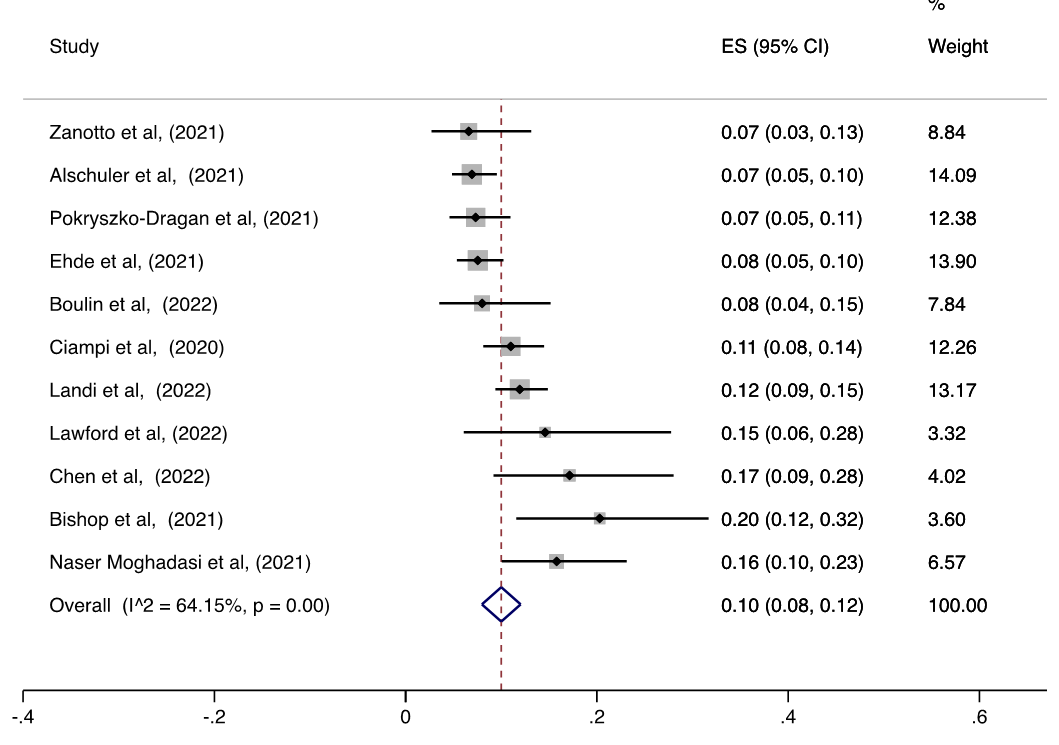

Supplement: Supplementary file 10 [file Image_9.TIF]

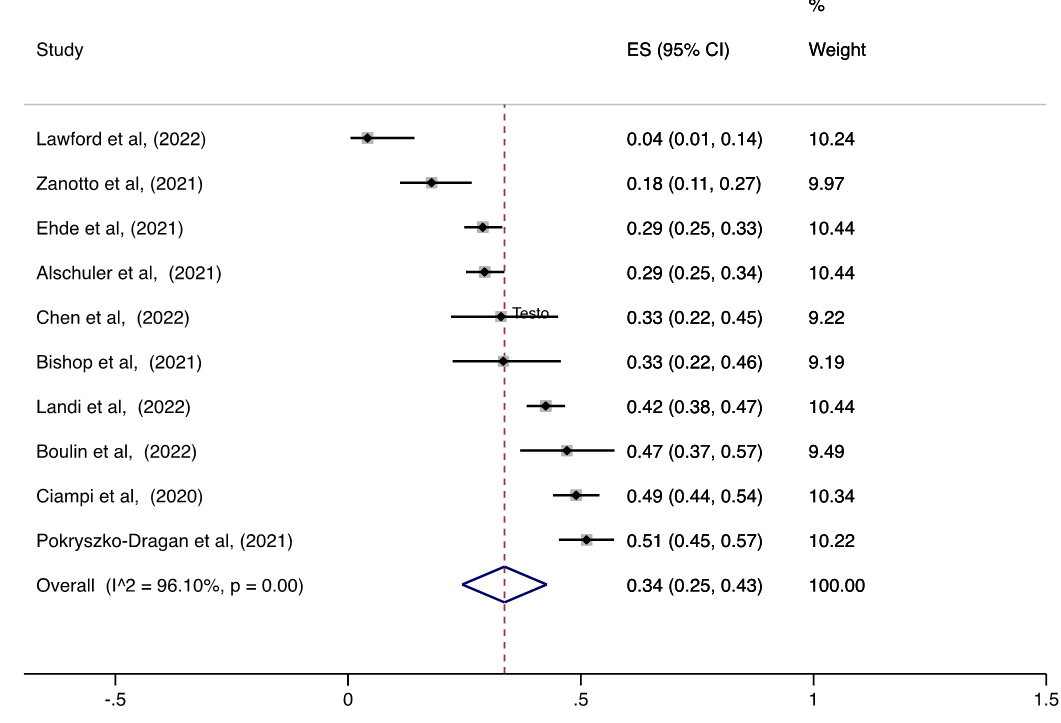

Supplement: Supplementary file 11 [file Image_10.TIF]

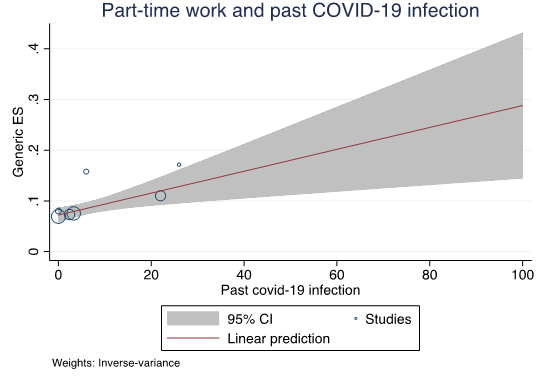

Supplement: Supplementary file 12 [file Image_11.TIF]

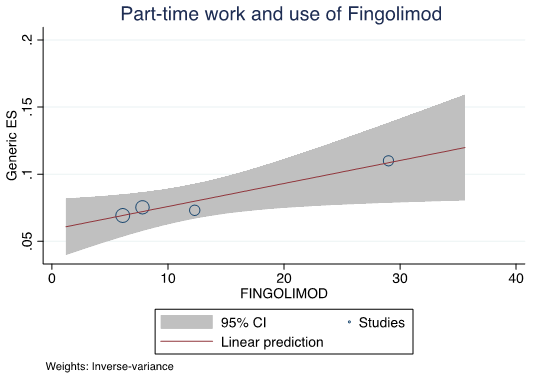

Supplement: Supplementary file 13 [file Image_12.TIF]

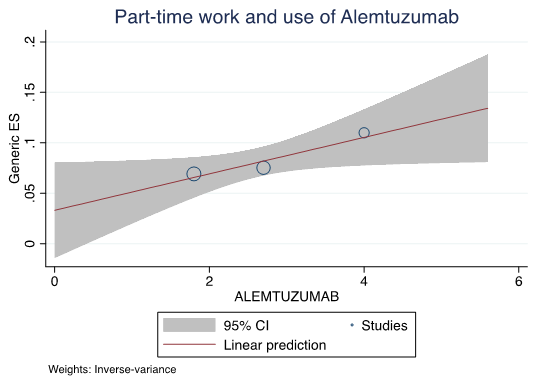

Supplement: Supplementary file 14 [file Image_13.PNG]
